# 放疗联合免疫治疗和化疗改善广泛期小细胞肺癌预后并展现协同作用

**DOI:** 10.3779/j.issn.1009-3419.2024.102.41

**Published:** 2024-11-20

**Authors:** Huaijun JI, Meiling SUN, Jingyi LI, Ge YU, Yongbing CHEN

**Affiliations:** ^1^215000 苏州，苏州大学附属第二医院胸外科（季怀君，陈勇兵）; ^1^Department of Thoracic Surgery, The Second Affiliated Hospital of Soochow University, Suzhou 215000, China; ^2^264200 威海，山东大学附属威海市立医院胸外科（季怀君，于戈）; ^2^Department of Thoracic Surgery; ^3^264200 威海，山东大学附属威海市立医院呼吸与危重症医学科（孙美玲）; ^3^Department of Respiratory and Critical Care Medicine; ^2^264200 威海，山东大学附属威海市立医院肿瘤放疗科（李静宜）; ^4^Department of Radiation Oncology, Weihai Municipal Hospital, Cheeloo College of Medicine, Shandong University, Weihai 264200, China

**Keywords:** 肺肿瘤, 免疫治疗, 放疗, 协同效应, Lung neoplasms, Immunotherapy, Radiotherapy, Synergistic effect

## Abstract

**背景与目的:**

广泛期小细胞肺癌（extensive-stage small cell lung cancer, ES-SCLC）是一种具有极强增殖和侵袭能力的恶性肿瘤。由于缺乏有效的综合治疗手段，ES-SCLC患者的临床预后较差。本研究旨在探讨放疗（radiotherapy, RT）联合免疫治疗（immunotherapy, IT）及化疗（chemotherapy, CT）对ES-SCLC患者的疗效及其协同作用。

**方法:**

回顾性分析145例接受一线CT治疗的ES-SCLC患者，采用Kaplan-Meier法和Log-rank检验进行生存分析，并通过倾向性评分匹配（propensity score matching, PSM）减少干扰因素。

**结果:**

所有患者的中位总生存期（median overall survival, mOS）和中位无进展生存期（median progression-free survival, mPFS）分别为15.7和6.9个月。IT+CT组较CT组的mOS显著延长（17.2 vs 13.5个月，P=0.047），RT+CT组的mOS（18.5 vs 12.3个月，P<0.001）和mPFS（7.1 vs 6.2个月，P=0.006）较CT组均显著改善。多因素分析显示，RT、IT及东部肿瘤协作组体能状态（Eastern Cooperative Oncology Group performance status, ECOG PS）评分是mOS的独立预测因素（P<0.05），性别及ECOG PS评分是mPFS的独立预测因素（P<0.05）。PSM后，RT+CT组的mOS（18.0 vs 12.1个月，P<0.001）和mPFS（7.1 vs 5.5个月，P=0.037）仍显著优于CT组。RT+IT+CT组的mOS较IT+CT组进一步延长（28.5 vs 15.8个月，P=0.017）。3-4级不良事件发生率为27.6%，未见5级不良事件。

**结论:**

RT联合IT及CT可显著改善ES-SCLC患者预后，且RT在协同作用中发挥关键作用，安全性良好，值得进一步研究与临床应用。

## Abstract (en)

**Background and objective:**

Extensive-stage small cell lung cancer (ES-SCLC) is a malignant tumor with remarkable proliferative and invasive ability, which has very poor clinical prognosis due to lack of effective treatments. This study aims to evaluate the efficacy and synergistic effects of radiotherapy (RT) combined with immunotherapy (IT) and chemotherapy (CT) in patients with ES-SCLC.

**Methods:**

A retrospective analysis was performed on 145 ES-SCLC patients treated with first-line CT. Kaplan-Meier analysis and Log-rank tests were used to evaluate survival outcomes, while propensity score matching (PSM) was applied to reduce confounding factors.

**Results:**

The median overall survival (mOS) and median progression-free survival (mPFS) for the entire cohort were 15.7 and 6.9 mon, respectively. The IT+CT group had a significantly longer mOS compared to the CT group (17.2 vs 13.5 mon, P=0.047). Similarly, the RT+CT group demonstrated superior mOS (18.5 vs 12.3 mon, P<0.001) and mPFS (7.1 vs 6.2 mon, P=0.006) compared to the CT group. Multivariate analysis identified RT, IT, and Eastern Cooperative Oncology Group performance status (ECOG PS) as independent prognostic factors for mOS (P<0.05), while gender and ECOG PS were independent predictors for mPFS (P<0.05). Following PSM, the RT+CT group continued to exhibit significant advantages in mOS (18.0 vs 12.1 mon, P<0.001) and mPFS (7.1 vs 5.5 mon, P=0.037) compared to the CT group. Notably, the RT+IT+CT group achieved a markedly longer mOS than the IT+CT group (28.5 vs 15.8 mon, P=0.017). Grade 3-4 adverse events occurred in 27.6% of patients, with no grade 5 adverse events reported.

**Conclusion:**

The combination of RT, IT, and CT significantly enhances the prognosis of ES-SCLC patients. RT plays a key role in their synergistic effects and demonstrates good safety, warranting further research and clinical application.

肺癌是中国以及世界上患病率和死亡率最高的恶性肿瘤^[1]^。其中，小细胞肺癌（small cell lung cancer, SCLC）占肺癌总数的13%-17%，其特点是快速增殖，早期易发生远处转移^[2]^。SCLC分为广泛期SCLC（extensive-stage small cell lung cancer, ES-SCLC）和局限期SCLC（limited-stage small cell lung cancer, LS-SCLC），临床上患者初诊时约70%已经处于广泛期，预后极差，传统治疗方案是以化疗（chemotherapy, CT）为基础的综合治疗，5年生存率不足7%^[3]^。

近年来，随着免疫治疗（immunotherapy, IT）的发展，为攻克ES-SCLC长久以来的治疗瓶颈带来了希望。研究^[4⇓-6]^显示程序性细胞死亡受体1（programmed cell death 1, PD-1）/程序性细胞死亡配体1（programmed cell death ligand 1, PD-L1）抑制剂联合CT可延长ES-SCLC患者的总生存期（overall survival, OS）和无进展生存期（progression-free survival, PFS）。此外，胸部放疗（thoracic radiotherapy, TRT）的加入改善了ES-SCLC局部控制率及预后^[7,8]^。在放疗（radiotherapy, RT）和IT协同机制作用下，RT能否为SCLC的治疗带来突破性进展，目前尚无定论。本研究旨在探讨现实世界中IT、RT联合CT治疗ES-SCLC患者的临床疗效和安全性，以及IT与RT是否能够协同增效。

## 1 资料与方法

### 1.1 病例选择

我们回顾性收集2016年4月至2023年8月在威海市立医院接受一线CT的ES-SCLC患者的病历资料。采集患者的基本信息，所有数据均来自临床医疗记录，随访从诊断之日起至全因死亡之日或失访者末次随访时间。本研究已通过威海市立医院伦理委员会审核批准（批准号：20240110）。

### 1.2 纳入与排除标准

（1）纳入标准：年龄在18岁或以上；东部肿瘤协作组体能状态（Eastern Cooperative Oncology Group performance status, ECOG PS）评分0-2分；病理诊断为SCLC，并且接受至少2个周期的CT；接受IT的患者至少完成2个周期；接受RT的患者需包括TRT。（2）排除标准：无明确的病理诊断；同时患有其他部位的肿瘤；ECOG PS评分>2分；CT或IT不足2个周期；接受RT的患者没有行TRT；存在自身免疫性疾病；治疗后无疗效评价或随访失败。

### 1.3 疗效评价

根据实体瘤疗效评估标准1.1版（Response Evaluation Criteria in Solid Tumours 1.1, RECIST 1.1），基于影像学检查评估对治疗的反应。主要终点是OS，其定义为从首次接受CT之日开始到全因死亡的时间，或最后一次随访之日。次要终点为PFS，定义为从首次接受CT之日开始到肿瘤进展、死亡或最后一次随访时间。根据国家癌症研究所不良事件通用术语标准5.0版评估不良反应。通过查阅住院记录、门诊病历和电话对患者进行随访。最后一次随访日期为2024年3月30日。

### 1.4 统计学方法

患者基线特征采用例数（%）表示，使用卡方检验做组间比较。按照1:1匹配比例和0.02匹配容差行倾向评分匹配（propensity score matching, PSM），匹配协变量包括：年龄、性别、吸烟史、ECOG PS评分、脑转移状况、肝转移状况、骨转移状况、肺转移状况、肾上腺转移状况、抗血管治疗、IT和RT状况。通过Kaplan-Meier法进行生存分析，应用Log-rank检验分析生存差异；采用Cox比例风险回归模型进行预后的单因素及多因素分析，单因素分析结果处于临界值的变量也纳入多因素分析。所有统计分析均使用SPSS 27.0版本进行。以P<0.05为差异具有统计学意义。

## 2 结果

### 2.1 一般资料

本研究筛选了385例患者，131例LS-SCLC患者被排除，31例患者因CT或IT不足2个周期被排除，接受RT的患者中15例因没有接受TRT被排除；23例患者因非一线接受IT被排除，40例患者因OS或PFS不能明确被排除。最终有145例符合条件的患者被纳入分析。中位年龄为65（44-85）岁，以男性为主（78.6%），ECOG PS评分0-1分人群占总数的93.1%。CT联合IT占34.5%，CT联合RT占52.4%。见表1、图1。

**表1:** PSM匹配前后一线治疗ES-SCLC患者一般临床资料比较 [n(%)]

Characteristics	Before PSM					After PSM (IT)					After PSM (RT)			
	Total (n=145)	CT+IT (n=50)	CT (n=95)	P		Total (n=84)	CT+IT (n=42)	CT (n=42)	P		Total (n=110)	CT+RT (n=55)	CT (n=55)	P
Age (yr)				0.472					0.381					>0.999
≤65	84 (57.9)	31 (62.0)	53 (55.8)			46 (54.8)	25 (59.5)	21 (50.0)			64 (58.2)	32 (58.2)	32 (58.2)	
>65	61 (42.1)	19 (38.0)	42 (44.2)			38 (45.2)	17 (40.5)	21 (50.0)			46 (41.8)	23 (41.8)	23 (41.8)	
Gender				0.066					0.287					0.621
Female	31 (21.4)	15 (30.0)	16 (16.8)			18 (21.4)	11 (26.2)	7 (16.7)			20 (18.2)	9 (16.4)	11 (20.0)	
Male	114 (78.6)	35(70.0)	79 (83.2)			66 (78.6)	31 (73.8)	35 (83.3)			90 (81.8)	46 (83.6)	44 (80.0)	
Smoking history				>0.999					>0.999					0.554
Never	58 (40.0)	20 (40.0)	38 (40.0)			32 (38.1)	16 (38.1)	16 (38.1)			41 (37.3)	19 (34.5)	22 (40.0)	
Former/current	87 (60.0)	30 (60.0)	57 (60.0)			52 (61.9)	26 (61.9)	26 (61.9)			69 (62.7)	36 (65.5)	33 (60.0)	
Family tumor history				0.038					0.124					>0.999
No	110 (75.9)	43 (86.0)	67 (70.5)			64 (76.2)	35 (83.3)	29 (69.0)			82 (74.5)	41 (74.5)	41 (74.5)	
Yes	35 (24.1)	7 (14.0)	28 (29.5)			20 (23.8)	7 (16.7)	13 (31.0)			28 (25.5)	14 (25.5)	14 (25.5)	
Hypertension				0.562					>0.999					0.844
No	94 (64.8)	34 (68.0)	60 (63.2)			54 (64.3)	27 (64.3)	27 (64.3)			69 (62.7)	34 (61.8)	35 (63.6)	
Yes	51 (35.2)	16 (32.0)	35 (36.8)			30 (35.7)	15 (35.7)	15 (35.7)			41 (37.3)	21 (38.2)	20 (36.4)	
Diabetes				0.356					>0.999					>0.999
No	122 (84.1)	44 (88.0)	78 (82.1)			72 (85.7)	36 (85.7)	36 (85.7)			92 (83.6)	46 (83.6)	46 (83.6)	
Yes	23 (15.9)	6 (12.0)	17 (17.9)			12 (14.3)	6 (14.3)	6 (14.3)			18 (16.4)	9 (16.4)	9 (16.4)	
ECOG PS				0.757					0.645					>0.999
0-1	135 (93.1)	47 (94.0)	88 (92.6)			79 (94.0)	40 (95.2)	39 (92.9)			104 (94.5)	52 (94.5)	52 (94.5)	
2	10 (6.9)	3 (6.0)	7 (7.4)			5 (6.0)	2 (4.8)	3 (7.1)			6 (5.5)	3 (5.5)	3 (5.5)	
Brain metastasis				0.131					>0.999					>0.999
No	112 (77.2)	35 (70.0)	77 (81.1)			66 (78.6)	33 (78.6)	33 (78.6)			92 (83.6)	46 (83.6)	46 (83.6)	
Yes	33 (22.8)	15 (30.0）	18 (18.9)			18 (21.4)	9 (21.4)	9 (21.4)			18 (16.4)	9 (16.4)	9 (16.4)	
Liver metastasis				0.081					0.306					0.815
No	116 (80.0)	36 (72.0)	80 (84.2)			64 (76.2)	34 (81.0)	30 (71.4)			87 (79.1)	43 (78.2)	44 (80.0)	
Yes	29 (20.0)	14 (28.0)	15 (15.8)			20 (23.8)	8 (19.0)	12 (28.6)			23 (20.9)	12(21.8)	11 (20.0)	
Bone metastasis				0.470					0.474					0.662
No	104 (71.7)	34 (68.0)	70 (73.7)			59 (70.2)	31 (73.8)	28 (66.7)			82 (74.5)	42 (76.4)	40 (72.7)	
Yes	41 (28.3)	16 (32.0)	25 (26.3)			25 (29.8)	11 (26.2)	14 (33.3)			28 (25.5)	13 (23.6)	15 (27.3)	
Lung metastasis				0.464					0.434					>0.999
No	117 (80.7)	42 (84.0)	75 (78.9)			65 (77.4)	34 (81.0)	31 (73.8)			90 (81.8)	45 (81.8)	45 (81.8)	
Yes	28 (19.3)	8 (16.0)	20 (21.1)			19 (22.6)	8 (19.0)	11 (26.2)			20 (18.2)	10 (18.2)	10 (18.2)	
Adrenal gland metastasis				0.199					0.236					0.463
No	131 (90.3)	43 (86.0)	88 (92.6)			77 (91.7)	37 (88.1)	40 (95.2)			102 (92.7)	52 (94.5)	50 (90.1)	
Yes	14 (9.7)	7 (14.0)	7 (7.4)			7 (8.3)	5 (11.9)	2 (4.8)			8 (7.3)	3 (5.5)	5 (9.9)	
Anti-angiogenic				0.160					0.663					>0.999
Yes	61 (42.1)	25 (50.0)	36 (37.9)			42 (50.0)	20 (47.6)	22 (52.4)			46 (41.8)	23 (41.8)	23 (41.8)	
No	84 (57.9)	25 (50.0)	59 (62.1)			42 (50.0)	22 (52.4)	20 (47.6)			64 (58.2)	32 (58.2)	32 (58.2)	
Radiotherapy				0.942					0.827					
Yes	76 (52.4)	26 (52.0)	50 (52.6)			41 (48.8)	21 (50.0)	20 (47.6)						
No	69 (47.6)	24 (48.0)	45 (47.4)			43 (51.2)	21 (50.0)	22 (52.4)						
Immunotherapy														0.675
Yes											32 (29.1)	17 (30.9)	15 (27.3)	
No											78 (70.9)	38 (69.1)	40 (72.7)	

ES-SCLC: extensive-stage small cell lung cancer; PSM: propensity score matching; RT: radiotherapy; IT: immunotherapy; CT: chemotherapy; ECOG PS: Eastern Cooperative Oncology Group performance status.

**图1:**
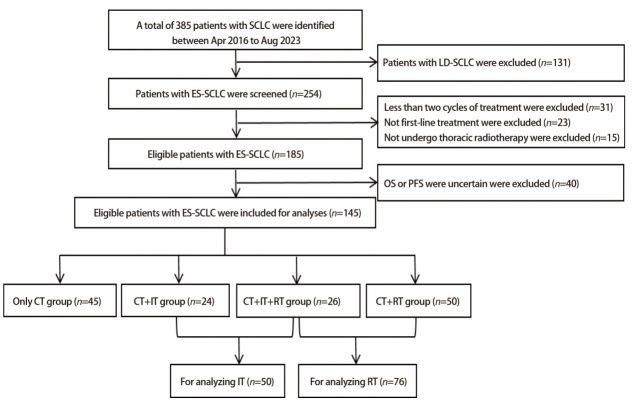
ES-SCLC患者筛选入组流程图

### 2.2 治疗方案

#### 2.2.1 CT

137例患者的CT方案是卡铂（n=52）或顺铂（n=85）联合依托泊苷治疗；6例患者接受了奈达铂联合依托泊苷治疗，2例患者接受了顺铂联合伊立替康治疗。

#### 2.2.2 IT

27例患者接受PD-1治疗（替雷利珠单抗13例，斯鲁利单抗5例，卡瑞利珠单抗3例，信迪利单抗2例，帕博利珠单抗2例，特瑞普利单抗1例，派安普利单抗1例），23例患者接受PD-L1治疗（度伐利尤单抗16例，阿替利珠单抗5例，阿得贝利单抗2例）。

#### 2.2.3 RT

放射治疗技术由放疗科医生根据中国临床肿瘤学会（Chinese Society of Clinical Oncology, CSCO）指南或美国国立综合癌症网络（National Comprehensive Cancer Network, NCCN）指南决定。调强放射治疗（intensity-modulated radiation therapy, IMRT）被选为放射治疗技术。计划靶区（planning target volume, PTV）和危及器官（organ at risk, OAR）是根据肺癌放射治疗指南制定的。临床靶区（clinical target volume, CTV）包括原发肿瘤部位和治疗后的阳性淋巴结。PTV为CTV外扩0.8 cm。总放射剂量30-60 Gy，中位放射剂量为45 Gy、2 Gy/F或3 Gy/F、5 F/W。为确保放射治疗的准确性，PTV大于95%处方剂量。危及器官限量具体为：脊髓Dmax<45 Gy；肺Dmean<17 Gy，全肺V5<60%，V20<30%，V30<20%；心脏Dmean<20 Gy，V30<40%，V40<30%；食管Dmean<30 Gy，V60<17%。76例患者均接受了原发灶的RT，其中9例患者联合颅脑转移灶RT，2例患者联合骨转移灶RT。

### 2.3 疗效评价

#### 2.3.1 OS和PFS比较

所有患者的中位随访时间为22.7个月。中位OS（median OS, mOS）为15.7个月（95%CI: 14.0-17.4）。1年OS率为67.4%，2年OS率为28.9%，5年OS率为5.6%。随访期间，118例（81.4%）患者达到主要终点，所有患者均出现复发。CT+IT组mOS较CT组显著延长[17.2（95%CI: 14.7-19.7） vs 13.5个月（95%CI: 11.0-16.0）；P=0.047]；总人群中位PFS（median PFS, mPFS）为6.9个月，两组无明显差异（7.1 vs 6.5个月，P=0.332）。此外，76例（52.4%）患者接受了CT+RT，69例（47.6%）患者没有接受RT（为CT），CT+RT组mOS为18.5个月（95%CI: 12.5-24.5），mPFS为7.1个月（95%CI: 6.6-7.6），均长于CT组的12.3（95%CI: 10.8-13.8）和6.2个月（95%CI: 4.9-7.5），差异均有统计学意义（P<0.001, P=0.006）。见图2。

**图2:**
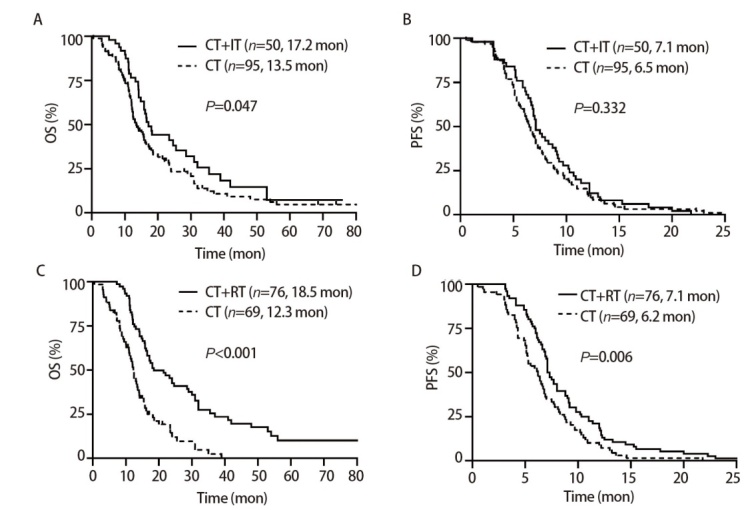
ES-SCLC患者的生存分析。A：CT+IT组和CT组OS的Kaplan-Meier曲线；B：CT+IT组和CT组PFS的Kaplan-Meier曲线；C：CT+RT组和CT组OS的Kaplan-Meier曲线；D：CT+RT组和CT组PFS的Kaplan-Meier曲线。

#### 2.3.2 单因素和多因素生存分析

单因素分析显示性别（P=0.010）、吸烟史（P=0.016）、ECOG PS评分（P=0.041）和RT（P<0.001）是影响OS预后的重要因素，IT的P值处于临界值（P=0.051）；性别（P=0.022）、ECOG PS评分（P=0.035）和RT（P=0.007）是影响PFS预后的重要因素。进一步行多因素分析显示，ECOG PS评分（HR=0.414, 95%CI: 0.199-0.858, P=0.018）、RT（HR=0.376, 95%CI: 0.255-0.555, P<0.001）和IT（HR=0.632, 95%CI: 0.420-0.950, P=0.027）是OS的独立预测因素；性别（HR=0.620, 95%CI: 0.402-0.958, P=0.031）和ECOG PS评分（HR=0.460, 95%CI: 0.231-0.917, P=0.027）是PFS的独立预测因素。见表2。

**表2:** 单因素和多因素Cox比例风险模型分析OS和PFS的潜在危险因素

Characteristics	OS						PFS				
	Univariate analysis			Multivariate analysis			Univariate analysis			Multivariate analysis	
	HR (95%CI)	P		HR (95%CI)	P		HR (95%CI)	P		HR (95%CI)	P
Age (≤65 vs >65 yr)	0.801 (0.555-1.156)	0.236					0.838 (0.601-1.169)	0.298			
Gender (Female vs Male)	0.536 (0.333-0.861)	0.010		0.656 (0.358-1.202)	0.172		0.631 (0.418-0.952)	0.022		0.620 (0.402-0.958)	0.031
Smoking history (Never vs Former/current)	0.632 (0.435-0.919)	0.016		0.726 (0.470-1.121)	0.148		0.785 (0.561-1.099)	0.158			
Family tumor history (No vs Yes)	1.062 (0.691-1.632)	0.784					1.180 (0.787-1.769)	0.423			
Hypertension (No vs Yes)	0.991 (0.678-1.449)	0.964					1.074 (0.760-1.518)	0.686			
Diabetes (No vs Yes)	1.009 (0.611-1.668)	0.971					0.680 (0.433-1.068)	0.094			
ECOG PS (0-1 vs 2)	0.507 (0.265-0.973)	0.041		0.414 (0.199-0.858)	0.018		0.497 (0.260-0.952)	0.035		0.460 (0.231-0.917)	0.027
Brain metastatic (No vs Yes)	1.283 (0.814-2.028)	0.281					0.907 (0.614-1.342)	0.626			
Liver metastatic (No vs Yes)	0.761 (0.491-1.181)	0.223					0.784 (0.520-1.181)	0.245			
Bone metastatic (No vs Yes)	0.686 (0.462-1.018)	0.061					1.006 (0.699-1.448)	0.974			
Lung metastatic (No vs Yes)	0.766 (0.484-1.213)	0.256					0.867 (0.568-1.322)	0.507			
Adrenal gland metastasis (No vs Yes)	1.434 (0.723-2.847)	0.303					0.744 (0.427-1.295)	0.296			
Anti-angiogenic (Yes vs No)	0.921 (0.637-1.329)	0.661					0.779 (0.557-1.090)	0.144			
Radiotherapy (Yes vs No)	0.371 (0.252-0.545)	<0.001		0.376 (0.255-0.555)	<0.001		0.634 (0.454-0.883)	0.007		0.722 (0.512-1.019)	0.064
Immunotherapy (Yes vs No)	0.674 (0.454-1.001)	0.051		0.632 (0.420-0.950)	0.027		0.845 (0.598-1.193)	0.338			

CI: confidence interval; HR: hazard ratio.

#### 2.3.3 PSM匹配后的比较

为了尽量减少组间混杂因素的干扰，而采用PSM。在CT+IT组与CT组中共有42对完成了匹配，CT+IT组mOS较CT组显著延长[16.6（95%CI: 13.4-19.8） vs 12.2个月（95%CI: 10.5-13.9）,P=0.005]；在mPFS方面两组无显著差异（7.1 vs 6.1个月，P=0.149）。同样，在CT+RT组与CT组中共有55对完成了匹配，CT+RT组mOS为18.0个月（95%CI: 12.1-23.9），mPFS为7.1个月（95%CI: 6.7-7.5），均长于CT组的12.1（95%CI: 10.8-13.4）和5.5个月（95%CI: 4.5-6.5），差异均有统计学意义（P<0.001, P=0.037）。见图3。

**图3:**
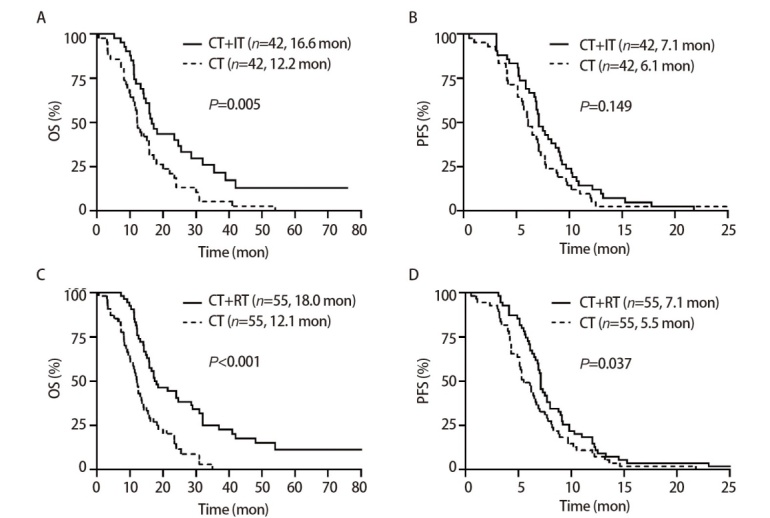
PSM后EC-SCLC患者的生存分析。A：CT+IT组和CT组OS的Kaplan-Meier曲线；B：CT+IT组和CT组PFS的Kaplan-Meier曲线；C：CT+RT组和CT组OS的Kaplan-Meier曲线；D：CT+RT组和CT组PFS的Kaplan-Meier曲线。

#### 2.3.4 根据RT和IT分为4组后的生存分析

我们将所有患者分为4组：RT+IT+CT组26例，RT+CT组50例，IT+CT组24例，CT组45例，mOS分别为28.5、18.0、15.8和11.2个月。与CT组相比，3组患者的OS均有显著改善（P<0.001, P<0.001, P=0.013），RT+IT+CT组较IT+CT组OS有明显改善（P=0.017）。4组mPFS分别为7.1、7.3、6.9、5.3个月，较CT组均有明显的PFS获益（P=0.006, P=0.001, P=0.037），3组间PFS无统计学差异。见表3、图4。

**表3:** 分析4组的生存结果

Group	n	OS					PFS			
		mOS (mon)	P_RT+CT_	P_IT+CT_	P_CT_		mPFS (mon)	P_RT+CT_	P_IT+CT_	P_CT_
RT+IT+CT	26	28.5	0.359	0.017	<0.001		7.1	0.765	0.932	0.006
RT+CT	50	18.0		0.087	<0.001		7.3		0.504	0.001
IT+CT	24	15.8			0.013		6.9			0.037
CT	45	11.2					5.3			

**图4:**
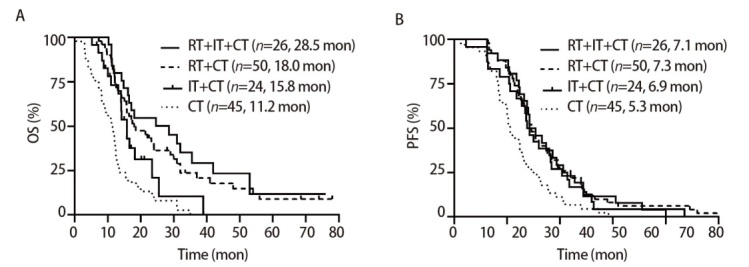
根据RT和IT分成4组后的患者生存分析。A：4组OS的Kaplan-Meier曲线；B：4组PFS的Kaplan-Meier曲线。

### 2.4 治疗相关不良事件

在所有入组的患者中，112例（77.2%）经历了至少一次治疗相关不良事件，其中72例（49.7%）为1-2级，40例（27.6%）为3-4级。其中11例患者发生3级肺炎，发生率为7.6%；4例患者发生3级食管炎，发生率为2.8%。没有出现5级不良事件。常见不良反应有乏力、厌食、恶心、白细胞减少、中性粒细胞减少等，对症处理后均能控制。

## 3 讨论

ES-SCLC特点是预后极差和治疗选择有限，传统的一线标准治疗方案是依托泊苷联合铂类化疗。虽然患者初始治疗客观缓解率可达50%-70%，但由于治疗后快速耐药，中位OS不足1年^[3]^。免疫治疗的发展让我们看到了ES-SCLC患者治疗的新希望^[9]^。大量的临床试验比如CASPIAN^[4]^、IMpower-133^[5]^和ASTRUM-005^[6]^，已经探索了PD-1/PD-L1抑制剂在治疗SCLC患者中的安全性，并显示出令人鼓舞的结果。基于这些临床研究，相关诊疗指南批准了IT联合CT作为ES-SCLC的一线标准治疗方案。然而，真实世界里IT在ES-SCLC患者中的临床获益仍值得进一步探索。

我们的研究表明，在总人群中，IT+CT组与单独CT组相比显著改善了OS（17.2 vs 13.5个月，P=0.047）。为排除干扰因素，我们进行了PSM，最终匹配了42对数据，进一步分析得到了相同的结果（16.6 vs 12.2个月，P=0.005），有力地证明了IT治疗的加入可延长ES-SCLC患者3.7-4.4个月的OS。然而在PFS方面，无论总人群（7.1 vs 6.5个月，P=0.332）还是PSM匹配后的人群（7.1 vs 6.1个月，P=0.149），IT+CT组与CT组相比PFS均未获益。这可能反映了免疫治疗的延迟效应和拖尾效应，免疫治疗起效缓慢，对于快速进展的ES-SCLC短期内控制能力有限，这些特点使得免疫治疗在长期生存上显示优势，而在早期疾病控制上未显著获益。既往研究如CASPIAN^[4]^（mPFS 5.1 vs 5.4个月）也报道了类似结果。另外，回顾性研究本身的局限性以及入组人数较少也可能是产生这种结果的干扰因素。值得注意的是，我们研究中患者OS在数值上优于所有已公布的一线III期研究结果，如CASPIAN^[4]^、IMpower-133^[5]^、ASTRUM-005^[6]^和CAPSTONE-1^[10]^研究（12.3-15.4个月）。同样，虽然我们的研究显示两组在PFS方面没有统计学差异，但是在数值上也超过了这些前瞻性研究的IT组（4.5-5.8个月）和CT组（4.3-5.6个月）。这考虑是由于在我们的研究中有超过一半（52.4%）的患者接受了RT。

研究显示，即使接受了标准传统治疗，75%-90%的患者仍有胸部病灶残留，胸部肿瘤进展是导致ES-SCLC患者死亡的主要原因^[7]^，RT作为一种有效的局部治疗手段在SCLC患者治疗中发挥重要作用。CT后巩固TRT可显著降低SCLC患者局部复发率，延长患者寿命，5年OS率提高8.7%（12.3% vs 3.6%, P<0.001）^[7,11]^。前瞻性研究和荟萃分析^[12,13]^表明，与单独CT相比，联合TRT提供了显著生存优势。我们的研究显示，在总人群和55对匹配后的人群中，RT均显著延长ES-SCLC患者的OS（18.5 vs 12.3个月，P<0.001；18.0 vs 12.1个月，P<0.001）和PFS（7.1 vs 6.2个月，P=0.006；7.1 vs 5.5个月，P=0.037）。这一发现与之前的研究一致，进一步证实了RT在ES-SCLC患者治疗中的地位。但是，并不是所有的患者都能从TRT中获益，一项回顾性研究^[14]^显示，与CT相比联合TRT并没有OS获益。

虽然RT或IT联合CT为ES-SCLC患者提供了更好的选择并改善了预后，OS可以提高2-5个月，但是预后仍然很差，5年生存率不足7%^[3]^，这就迫切地需要我们做更多的探索去提升这部分患者的预后。研究^[15,16]^表明RT可以通过增加肿瘤抗原暴露和调节性T细胞浸润重塑免疫微环境，从而增强IT的疗效，而IT又可以增强RT的远隔效应。Daher等研究^[17]^表明，IT联合CT后接受TRT的ES-SCLC患者OS（27.7 vs 13.2个月，P<0.007）和PFS（8.5 vs 5.6 个月，P<0.003）明显延长，且安全性良好，与我们的RT+IT+CT组mOS（28.5个月）非常接近，均达到了超长生存期。今年的欧洲肺癌大会（European Lung Cancer Conference, ELCC）上发表的LEAD研究强调了全身治疗与局部治疗相结合在临床实践中的可行性和潜在应用前景^[18]^。根据我们的亚组分析，RT+IT+CT组、RT+CT组、IT+CT组和CT组患者的mOS分别为28.5、18.0、15.8和11.2个月。IT+CT组较CT组有明显的OS获益（P=0.013），再进一步联合RT（RT+IT+CT）后较免疫组（IT+CT）OS又得到了明显提升（P=0.017），证实了RT和IT的联合应用在ES-SCLC的治疗中起到协同增效的作用。我们的研究在PFS方面，并没有得到明显获益，考虑可能的因素是ES-SCLC进展较快，而IT虽然有拖尾现象，但是在现实世界中相对起效比较慢；另外，不除外回顾性研究固有的局限性或者入组患者较少引起的这种现象。

一项回顾性研究^[19]^评估了在ES-SCLC患者中将IT与TRT结合使用的安全性和有效性，并揭示了TRT联合IT并没有表现出额外的不良事件。此外，TRT相关不良事件的发生与之前其他研究的发现一致，并且被认为是可以控制的。我们的研究虽然有77.2%的患者出现不良事件，但是大部分患者（49.7%）为1-2级的轻度不良事件，没有出现5级不良事件，验证了在ES-SCLC患者中将IT与RT联合使用的安全性。

本文的主要优势在于不仅证实了RT和IT在ES-SCLC患者中的有效性和安全性，而且进一步证实RT和IT在ES-SCLC患者中的协同作用。然而，我们的研究有一些局限性：首先，由于研究设计是单中心回顾性的，PSM匹配也不能完全排除混杂变量和选择偏倚；其次，虽然有超过一半的患者接受了RT，但除了TRT之外，还有少数患者行脑RT和骨RT，这难免也影响分析结果；第三，本研究的次要终点PFS定义的截止时间包括死亡，存在部分患者PFS不准确，可能会影响分析结果；最后，接受IT的患者数量较少，而且是使用了多种抗PD-1/PD-L1药物，可能会影响分析结果。因此，需要进一步的大规模真实世界研究来验证我们的结果。

本研究认为，虽然ES-SCLC患者的治疗选择越来越多，但CT依然是基石，IT或RT联合CT都可以延长ES-SCLC患者的生存期，具有一定协同增效作用，并且具有良好的安全性，值得临床进一步探索及应用。
